# Evaluation of the analgesic effect of vertebral cancellous bone infiltration anaesthesia during vertebroplasty

**DOI:** 10.1186/s13018-020-01872-7

**Published:** 2020-08-20

**Authors:** Zhaofei Zhang, Feng Jiao, Yonghui Feng, Chunliang Xie, Fengwei Qin, Sineng Zhang, Donghua Liu, Wang Tang

**Affiliations:** Department of Orthopedic Surgery, Guangzhou Hospital of Integrated Traditional and Western Medicine, 87 Yingbin Road, Huadu District, Guangzhou, 510800 Guangdong China

**Keywords:** Vertebroplasty, Vertebral cancellous bone infiltration anaesthesia, Local anaesthesia, VAS

## Abstract

**Objective:**

To evaluate the analgesic effect of vertebral cancellous bone infiltration anaesthesia during percutaneous vertebroplasty (PVP).

**Methods:**

Patients treated with vertebral cancellous bone infiltration anaesthesia (intervention group) or local anaesthesia alone (control group) during PVP at our institution during 2016–2018 were reviewed. The visual analogue scale (VAS) score before the operation, during establishment of the puncture channel, during pressure changes in the vertebral body (e.g., when removing or inserting pushers or needle cores), during bone cement injection, immediately after the operation, and at 2 h and 1 day postoperatively were compared between the groups. The patient’s satisfaction with the operation was recorded and compared between groups.

**Results:**

A total of 112 patients were enrolled (59 cases in the intervention group and 53 cases in the control group). There was no difference in the VAS score between the groups before the operation or during establishment of the intraoperative puncture channel (*P* > 0.05). The VAS score in the intervention group was significantly lower than that in the control group during pressure changes in the vertebral body (removal or insertion of puncture needle cores or pushers) and bone cement injection (*P* < 0.05). Immediately after the operation and at 2 h postoperatively, the pain in the intervention group was also significantly lower than that in the control group (*P* < 0.05), but there was no significant difference between the groups at 1 day postoperatively (*P* > 0.05). The patient satisfaction rate was 88% (52/59) in the intervention group and 67% (35/53) in the control group (*P* < 0.05).

**Conclusions:**

Vertebral cancellous bone infiltration anaesthesia may effectively relieve intraoperative pain and improve the surgical experience of patients without affecting the clinical effect of surgery.

## Introduction

Percutaneous vertebroplasty (PVP) has been widely used in the treatment of osteoporotic vertebral compression fractures (OVCFs); PVP can quickly relieve pain, allow early ambulation, and improve the quality of life of patients [1–4]. General or local anaesthesia can be used for this operation. In recent years, with the widening of indications for the implementation of general anaesthesia, its adverse reactions or complications have also been followed. General anaesthesia increases the incidence of cardiovascular and respiratory complications because basic diseases, such as those affecting the heart and lungs, are generally present in elderly patients, which increases the risk of anaesthesia and is not conducive to early recovery after surgery. Some researchers have confirmed that PVP is feasible under local anaesthesia alone [5]. It has relatively low risk, high safety and few complications. However, in the course of the operation, especially when the pressure changes in the vertebral body, such as during insertion or removal of the pushers and needle cores or during bone cement injection, the patients experience obvious pain, which causes them to have an extremely uncomfortable and fearful surgical experience. To obtain a more satisfactory analgesic effect for patients, we used vertebral cancellous bone infiltration anaesthesia to strengthen the analgesic effect, reduce intraoperative pain and improve the surgical experience.

## Materials and methods

### Selection of patients

A series of patients who underwent PVP for a single vertebral body between June 2016 and June 2018 in our hospital were retrospectively evaluated. The inclusion criteria were as follows: (1) a single vertebral body compression fracture; (2) vertebral cancellous bone infiltration anaesthesia or local infiltration anaesthesia; (3) bone mineral density (BMD) less than − 2.5; (4) PVP with a bipedicular approach; and (5) fracture without nerve or spinal cord compression symptoms. The exclusion criteria were as follows: (1) malignancy or angioma; (2) nonpainful OVCF; (3) burst fracture of vertebral body; and (4) multisegment vertebral fracture.

The patients who were finally included were divided into two groups according to the anaesthesia method. The intervention group consisted of those who underwent vertebral cancellous bone infiltration anaesthesia, and the control group consisted of those who underwent local infiltration anaesthesia alone.

### Surgical technique

**Fig. 1:**
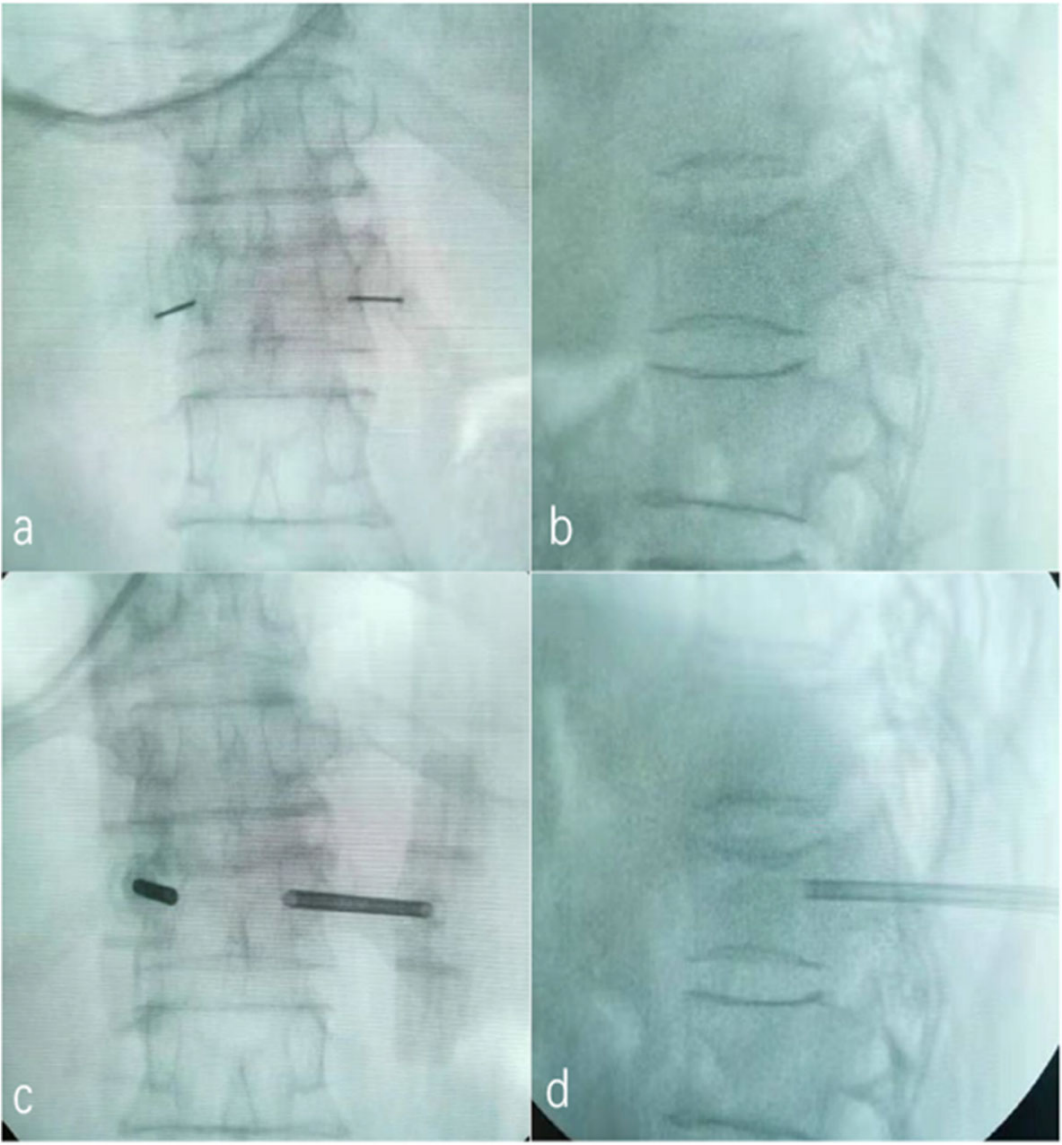
Local anaesthesia and vertebral cancellous bone infiltration anaesthesia procedure. **a** The location and depth of local anaesthesia on the anteroposterior film; **b** the location and depth of local anaesthesia on the lateral film; **c** the location and depth of vertebral cancellous bone infiltration anaesthesia on the anteroposterior film; **d** the location and depth of vertebral cancellous bone infiltration anaesthesia on the lateral film

All patients were placed in the prone position, and the pelvis and chest were elevated with a soft pillow to suspend the abdomen. The injured vertebral body was located using C-arm fluoroscopy, and the projection of the bilateral pedicles on the body surface were marked using a marker pen. Local infiltration anaesthesia with 1% lidocaine (approximately 8 ml on both sides) from shallow to deep was performed until the bone surface of the puncture area was reached. A sharp scalpel was used to cut the skin (approximately 5 mm) after successful administration of local anaesthesia. Then, the puncture needles (diameter, 3.0 mm (thoracic vertebrae) or 3.5 mm (lumbar vertebrae)) were inserted and pushed forward to enter the vertebral body (anterior pedicles) through points A, B and C [6]. After removing the needle cores, 0.05% lidocaine (a total of approximately 4 ml) was slowly injected into the vertebral body using a bipedicular approach (Fig. 1). Then, bone drills were used to drill holes in the vertebral body 3 min after the lidocaine was injected. Subsequently, sterile water and bone cement were fully mixed, and the bone cement was injected into the pushers (a pusher contained approximately 0.9 ml of bone cement). The pushers with bone cement were then inserted into the puncture channels to reach the area behind the front edge of the vertebral body. The bone cement was injected stepwise from the front to the rear until the cement reached the rear of the vertebral body (in front of the pedicles). Then, the pushers were removed, and the needle cores were installed. The needles were removed rotationally when the cement was hot. The incisions were sterilized with alcohol and then bandaged.

### Parameters observed

The patient’s information, including age, weight, height, BMD (T-score), days of hospitalization, operative duration, intraoperative blood loss volume, and injected cement volume, were collected.

The visual analogue scale (VAS) score was recorded as the observation index before the operation, during establishment of the intraoperative puncture channels, during pressure changes in the vertebral body (e.g., when removing or inserting pushers or needle cores), during bone cement injection, immediately after the operation, and at 2 h and 1 day postoperatively. The VAS score was repeatedly evaluated during pressure changes in the vertebral body and bone cement injection, and the highest score was finally retained. The patient’s satisfaction with the operation was reflected by whether they were willing to accept treatment with PVP again.

### Statistical analysis

Statistical analysis was performed using the SPSS 24.0 software (IBM Corporation, Armonk, New York, USA). Continuous data are expressed as the means ± standard deviation, and comparisons between the two groups were performed using the independent samples *t* test. Count data were compared using the chi-square test. The test level was *α* = 0.05.

## Results

A total of 112 patients treated with PVP, including 59 treated with vertebral cancellous bone infiltration anaesthesia and 53 treated with local anaesthesia alone, were enrolled in our study. We defined these 59 and 53 cases as the intervention and control groups, respectively. The cemented vertebral body levels were T_7_ to L_4_ in the two groups. The age, weight, height, BMD, days of hospitalization, intraoperative blood loss volume, and injected cement volume were not significantly different between the groups (*P* > 0.05) (Table 1). All patients underwent a smooth surgical procedure without significant adverse reactions or complications.

**Table 1:** Patient’s information

	Group A (*n* = 59)	Group B (*n* = 53)	*P* value
Patients (female/male)	33/26	33/20	0.496
Age (years)	74.68 ± 9.62	72.21 ± 9.30	0.171
Weight (kg)	60.78 ± 9.34	61.79 ± 12.44	0.625
Height (cm)	160.90 ± 9.58	162.28 ± 8.42	0.421
BMD (T score)	− 3.50 ± 0.86	− 3.34 ± 0.63	0.256
Hospital days (days)	3.73 ± 1.18	3.68 ± 1.54	0.848
Operation time (min)	32.19 ± 6.56	28.70 ± 8.31	0.015
Intraoperative blood loss (ml)	12.54 ± 4.26	13.38 ± 3.90	0.284
Injected cement volume (ml)	3.30 ± 0.72	3.45 ± 0.63	0.232

The operative duration in the intervention group was longer than that in the control group (*P* = 0.015). There was no significant difference in the VAS score between the groups during establishment of the intraoperative puncture channels (*P* = 0.762). During pressure changes in the vertebral body, especially when inserting or removing pushers or needle cores, the VAS score in the intervention group was significantly lower than that in the control group (*P* = 0.000). The VAS score in the intervention group was also lower than that in the control group during bone cement injection (*P* = 0.000), immediately after surgery (*P* = 0.045), and at 2 h postoperatively (*P* = 0.017). The pain in both groups was relieved at 1 day postoperatively compared with that before surgery, and the difference was not statistically significant between groups (*P* = 0.171) (Table 2). The patient satisfaction rate was 88% (52/59) in the intervention group and 67% (35/53) in the control group (*P* < 0.05).

**Table 2:** Comparisons of VAS scores at different time points between the two groups

Time points	Group A (*n* = 59)	Group B (*n* = 53)	*P* value
Pre-operation	7.88 ± 1.08	7.75 ± 1.09	0.539
Establishment of the puncture channel	6.27 ± 1.17	6.33 ± 1.20	0.762
Pressure changes in the vertebral body	6.86 ± 1.29	8.33 ± 1.30	0.000
Bone cement injection	7.11 ± 1.19	8.71 ± 1.08	0.000
Immediately after the operation	3.35 ± 1.06	3.75 ± 1.01	0.045
Postoperative 2 h	2.52 ± 1.08	3.00 ± 0.96	0.017
Postoperative 1 day	2.16 ± 0.83	2.39 ± 0.90	0.171

## Discussion

PVP has been widely used to treat OVCFs. It has the advantages of relieving pain, maintaining vertebral height, and reducing the duration of bedrest, thus avoiding some complications caused by bedrest, such as bedsores, pneumonia, deep vein thrombosis, and even stroke [7, 8]. The anaesthesia methods for PVP include local and general anaesthesia. The risk of general anaesthesia is high because cardiovascular and respiratory diseases are common in the elderly. Local anaesthesia is less risky during PVP, but pain during the procedure is evident. The main cause of pain may be that the anaesthetic infiltrates only the soft tissue and not the bone tissue. When the pressure changes in the vertebral body, the patient suffers severe pain. Accordingly, we performed vertebral cancellous bone infiltration anaesthesia, which relieves the patient’s pain caused by operating on the vertebral body, reduces the patient’s fear during PVP, and improves the patient’s surgical satisfaction. In our study, we significantly relieved patient pain using this anaesthesia method compared with traditional local anaesthesia alone. The incidence of intraoperative or postoperative complications was not increased, and the same clinical results after surgery were achieved.

Intraosseous anaesthesia was first reported by Orlov [9] in 1960 for plastic surgery of the hand. Waisman et al. [10] applied intraosseous local anaesthesia in 109 cases of surgery for limb fracture and obtained a satisfactory anaesthetic effect. Dauri et al. [11] compared intraosseous lidocaine alone and lidocaine with a sedative (remifentanil) and found a good analgesic effect in both groups. Although combined intravenous sedatives have a better analgesic effect during puncture, positioning and bone cement injection, they are often associated with a higher risk in elderly patients with OVCFs, especially in prone surgery. It is necessary to strictly grasp their indications after assessing systemic conditions and use them under monitoring. Therefore, intraosseous lidocaine is more suitable for elderly patients with OVCFs. Based on previous studies, we consider vertebral cancellous bone infiltration anaesthesia to be feasible.

Existing studies have shown that the pain caused by OVCFs may be related to the following factors: (1) microfracture of vertebral trabecular bone stimulates peripheral nerves, causing pain [12], and (2) vertebral fracture leads to decreased stability of the spine, causing damage to the lower back muscles and fascia [13, 14]. However, there is still controversy regarding the analgesic mechanism of PVP. Currently, the generally accepted analgesic mechanism may be related to the following factors [12, 15, 16]: (1) mechanical strength recovery and stable reconstruction; (2) thermal factors, such as thermal necrosis of nerve tissue and pain nociceptors caused by heat generated during bone cement aggregation; and (3) chemical factors, such as the concentration of instantaneously aggregated bone cement monomer producing toxicity and leading to the necrosis of nerve endings. The aggravation of pain during PVP may be associated with fracture reduction, pressure changes in the vertebral body, surgical procedures, or inflammatory reactions induced by bone cement stimulating nerve endings. The vertebral body is innervated by the sinuvertebral nerve of the spinal nerve and the sympathetic nerve fibres from the paravertebral nerve. Chandler et al. [17] reported that they used a terminal branch block of the sinuvertebral nerve to relieve pain in patients with OVCFs. In PVP, these nerve endings are stimulated to aggravate pain during surgical procedures, changes in pressure in the vertebral body, or inflammation caused by bone cement. For instance, when the operation is performed, the instability of these microfracture areas is aggravated, thereby stimulating nerve endings and causing pain. Studies have confirmed that when the pressure in the vertebral body increases, the pain is aggravated in patients with OVCFs [18]. Therefore, when puncture needle cores or pushers are inserted or removed, the pressure changes in the vertebral body, which may stimulate nerve endings, thereby causing severe pain for patients during PVP. In addition, the inflammatory reaction caused at the moment of bone cement injection also stimulates the nerve endings, causing obvious pain. Thus, vertebral cancellous bone infiltration anaesthesia is essential for relieving pain in patients during PVP. In our study, we confirmed that this anaesthesia method could significantly relieve pain in patients during intravertebral pressure changes and bone cement injection compared with local anaesthesia alone. This result is consistent with the results of Sesay [19], who believes that the basic mechanism of lidocaine involves the blocking of pain transmission from the local sinuvertebral nerve branches in the vertebral body.

Studies on venous angiography of the vertebral body have confirmed that the drainage vein develops slowly, and some of the vertebral body veins do not develop; therefore, after the injection of a nonionic contrast agent with high water solubility and low permeability into the vertebral body via the puncture needle, the contrast agent remains in the vertebral body for a long period of time [20]. Thus, the drug does not quickly drain from the venous system after entering the vertebral body through the puncture channel. These results explain why the VAS scores in the vertebral cancellous bone infiltration anaesthesia group were lower than those in the control group immediately after the operation and at 2 h postoperatively. Although vertebral cancellous bone infiltration anaesthesia allows a small amount of drug to directly enter the blood circulation, no cases of anaesthetic drug poisoning reaction were found in our study. Sesay et al. [19] collected venous blood samples from patients at several time points for lidocaine concentration detection after injecting 5 ml of 1% lidocaine into the vertebral body. The results showed that the highest blood drug level was still far below the level of toxicity, which confirmed the safety of the analgesic drug. We used a concentration of 0.05%, which not only further reduced the possibility of an anaesthetic poisoning reaction but also yielded an excellent anaesthetic effect.

Accurate puncture is essential for vertebral cancellous bone infiltration anaesthesia. An excessive puncture angle may pierce the medial wall of the pedicle such that the anaesthetic may enter the spinal canal through the damaged area, resulting in epidural anaesthesia, which brings unnecessary risk. Wang Song et al. [21] reported that in one case, the puncture needle was inserted into the spinal canal through the medial wall of the pedicle and punctured the dura mater and the arachnoid due to the excessive obliquity of the puncture angle. When the anaesthetic was injected into the working channel, which did not reach the vertebral body, the drug eventually mistakenly entered the subarachnoid space and caused a high level of spinal anaesthesia. Based on our previous study of three-point precision puncture [6], we can ensure that the working channel smoothly passes through the pedicle into the vertebral body, thus avoiding such risks. In addition, a preoperative CT examination is necessary. For fractures of the posterior wall of the vertebral body found on CT, it is necessary to be cautious when injecting anaesthetic drugs. During the vertebral cancellous bone infiltration anaesthesia procedure, the lidocaine should not be injected too quickly, as this may cause the lidocaine to enter the venous system rapidly and in large quantities, resulting in serious adverse reactions. We acknowledge that our study has several limitations. First, this was a single-centre study and lacked unbiased randomisation of samples. Second, the sample size was relatively small.

## Conclusions

In summary, vertebral cancellous bone infiltration anaesthesia may effectively relieve intraoperative pain and improve the surgical experience of patients while not affecting clinical outcomes.

## Data Availability

There is no other supporting data.

## Abbreviations

PVP: Percutaneous vertebroplasty
VAS: Visual analogue scale
OVCFs: Osteoporotic vertebral compression fractures
BMD: Bone mineral density
